# Modeling pollen-mediated gene flow from glyphosate-resistant to -susceptible giant ragweed (*Ambrosia trifida* L.) under field conditions

**DOI:** 10.1038/s41598-017-16737-z

**Published:** 2017-12-06

**Authors:** Zahoor A. Ganie, Amit J. Jhala

**Affiliations:** Department of Agronomy and Horticulture, University of Nebraska–Lincoln, Lincoln, 68583 Nebraska USA

## Abstract

A field experiment was conducted to quantify pollen mediated gene flow (PMGF) from glyphosate-resistant (GR) to glyphosate-susceptible (GS) giant ragweed under simulated field conditions using glyphosate resistance as a selective marker. Field experiments were conducted in a concentric design with the GR giant ragweed pollen source planted in the center and GS giant ragweed pollen receptors surrounding the center in eight directional blocks at specified distances (between 0.1 and 35 m in cardinal and ordinal directions; and additional 50 m for ordinal directions). Seeds of GS giant ragweed were harvested from the pollen receptor blocks and a total of 100,938 giant ragweed plants were screened with glyphosate applied at 2,520 g ae ha^−1^ and 16,813 plants confirmed resistant. The frequency of PMGF was fit to a double exponential decay model selected by information-theoretic criteria. The highest frequency of gene flow (0.43 to 0.60) was observed at ≤0.5 m from the pollen source and reduced rapidly with increasing distances; however, gene flow (0.03 to 0.04) was detected up to 50 m. The correlation between PMGF and wind parameters was inconsistent in magnitude, direction, and years.

## Introduction

Gene flow is the natural process of disseminating genetic information from one breeding population to another (usually) related population or between the closely related species^1^. More precisely, gene flow includes the incorporation of new genes into the gene pool^2^, or a change in the frequency of existing genes in a population^1,3^. Pollen-mediated gene flow (PMGF) is the movement of genes via pollen within and between populations of species of the same genetic background^4^. PMGF occurs in almost all flowering plant species due to the movement of pollen through wind, water, pollinators, or other means^1,3,5^. The frequency of PMGF depends on several factors, including the reproductive biology, breeding system, pollen viability, and pollen dispersal mechanism of a plant species, among other factors^6,7^. Furthermore, size, structure, and proximity among populations^8,9^ and environmental factors also play a significant role in PMGF^10,11^. Gene flow is considered a strong and dynamic evolutionary force that promotes evolution and speciation along with natural selection and influences the genetic diversity, adaptation, and fitness of a population^12–14^. In cases where natural selection and genetic drift are absent, gene flow promotes genetic homogeneity and maintains genetic cohesiveness in a population^5,15,16^.

Concerns related to gene flow in agriculture became prominently emphasized in both the public domain and scientific literature due to the development and commercialization of genetically-modified (GM) crops, which raised questions about the co-existence of GM and non-GM crops^3,17^. The major concern with GM crops is the escape of the transgene into either non-GM crops or closely related species^18–23^. Additional concerns with GM crops include the emergence of volunteers as weeds in subsequent crops such as glyphosate-resistant (GR) corn volunteers in GR soybean fields in the Midwest^24^, and the evolution of new invasive plants in natural habitats^25^. A rapid adoption of GM crops occurred with the commercialization of GR crops including soybean [*Glycine max* (L.) Merr.], corn (*Zea mays* L.), canola (*Brassica napus* L.), cotton (*Gossypium hirsutum* L.), and sugarbeet (*Beta vulgaris*)^26–28^. GR crops revolutionized weed management by permitting the in-crop use of glyphosate—a once in a century herbicide^29^. Glyphosate is effective on a wide spectrum of grasses and broadleaf weeds without the potential for carryover injury to crops grown in rotation^26,29,30^. Widespread adoption of GR crops primarily in the United States and Canada encouraged the use of conservation tillage, resulting in a considerable increase in the profitability of agronomic cropping systems^29^. However, glyphosate use has reduced the diversity of herbicides used for weed control, specifically in soybean and cotton^31,32^.

The overreliance on glyphosate for weed control in GR crops resulted in the evolution of GR  weeds^33^. As of December 2016, 35 weed species, including 16 grasses and 19 broadleaf weeds, have evolved resistance to glyphosate worldwide, including 16 species in the United States^33^. The evolution of GR weeds not only reduces weed control options and the utility of GR crops, but also has long-term ecological consequences such as shifts in weed species composition and the persistence of the resistance trait in agricultural ecosystems. In most GR weed species, the evolution of resistance is due to target and/or non-target site mechanisms controlled by a single dominant or semi-dominant gene with nuclear inheritance^34–36^. Thus, there exists the possibility of glyphosate resistance spreading through pollen movement, especially in cross-pollinated species^34^. Gene flow via pollen dispersal delivers an initial source of resistant alleles to a susceptible weed population at a higher rate compared to the hypothetical mutation rate (1 × 10^−6^ for a gamete at a locus per generation), resulting in the rapid evolution and dissemination of resistance genes in new areas^13,34^. PMGF from GM crops to conventional crops or their weedy and wild relatives has been extensively studied to understand the consequences of introducing domesticated alleles or transgenes in natural populations^5,11,37–41^; however, more scientific information is needed to understand the dissemination of herbicide resistant traits between biotypes of the same weed species or closely related species for improved management strategies^42–47^.

The fate of a resistant allele in a weed population is influenced by the heritability, fitness, and reproductive and gene dispersal systems of the resistant biotype^34,48^. PMGF is particularly important in weed species such as giant ragweed that are characterized by their outcrossing nature and restricted seed mobility due to their large seed size^49^. Giant ragweed is a competitive summer annual broadleaf weed found throughout the United States and southern Canada^50–52^. It is a monoecious species, meaning that separate male and female flowers are present on the same plant. The male flowers occur in the terminal recemes at the top of the plant and the female flowers occur in clusters at the axils below the male flowers^51,53^. The male flowers produce considerably more pollen grains than the female flowers need to pollinate on a single plant. During flowering period, a single giant ragweed plant can produce an estimated 10 million pollen grains daily and more than a billion pollen grains during its life cycle^51,53^. Exposure to giant ragweed pollen also causes allergic rhinitis and asthma^54^.

Giant ragweed’s excessive pollen production allows individual plants to cross-pollinate, leading to variations in physical appearance and genetic diversity, and consequently a greater potential for resistance genes to migrate through pollen movement^51^. Brabham *et al*.^50^ documented an outcrossing rate of 31% between GR and glyphosate-susceptible (GS) giant ragweed biotypes while growing side by side at a distance of 76 cm (row spacing) and also suggested that GR is expressed as a dominant phenotype in giant ragweed. However, scientific literature is not available on how far a viable pollen can move and the frequency of gene flow in giant ragweed and role of wind direction. The objectives of this study were to determine the PMGF between GR and GS giant ragweed biotypes under simulated field conditions, and to understand the potential role of physical distance, wind speed, and wind direction in the dissemination of the glyphosate resistance trait in giant ragweed.

## Results

### Meteorological Data

**Figure 1:**
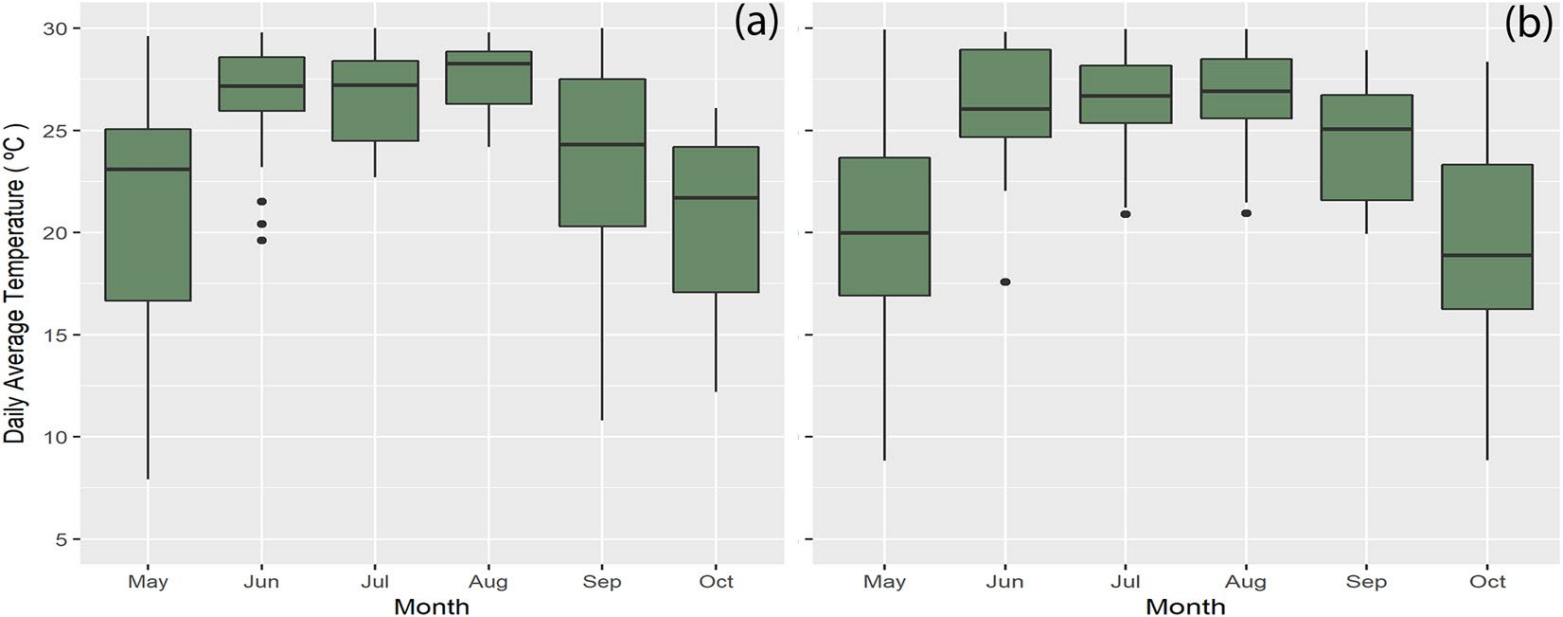
Daily average air temperature (C) from May to October in (**a**) 2014 and (**b**) 2015 at the South Central Agricultural Laboratory (SCAL), Clay Center, NE. The boxplots show the variation in daily average temperature (°C) for each month during which field studies were conducted in 2014 and 2015.

**Figure 2:**
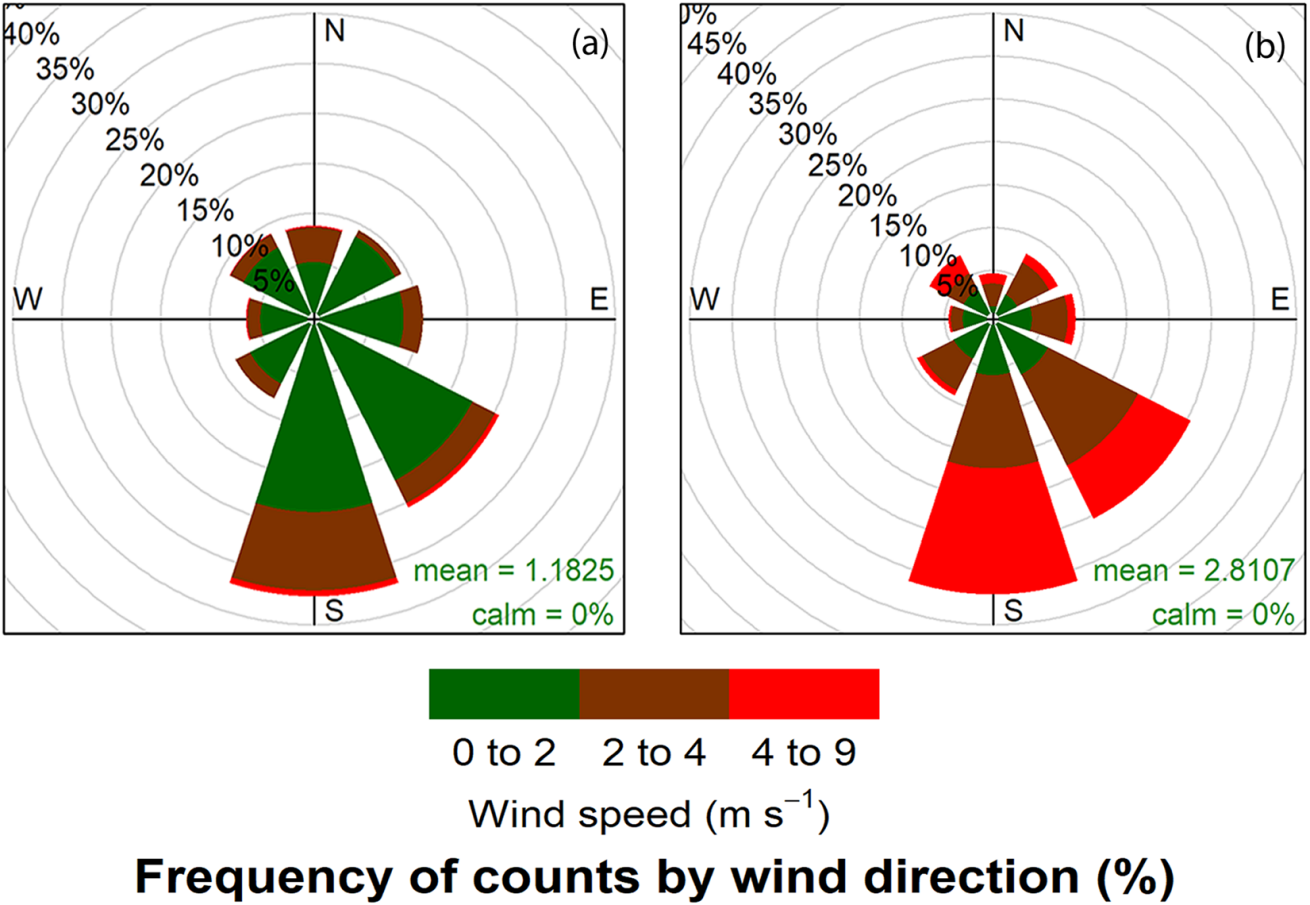
Wind rose plots displaying wind speed (m s^−1^) and wind frequency (%) in four cardinal (N, S, E, W) and four ordinal (NE, NW, SE, SW) directions during the flowering period for giant ragweed in (**a**) 2014 and (**b**) 2015 at the experimental site at South Central Agricultural Laboratory (SCAL), Clay Center, NE. The plots show the direction from which the wind was blowing in a particular year.

Mean monthly air temperature during the growing season varied from 14 °C to 24 °C and 15 °C to 25 °C in 2014 and 2015, respectively (Table S1). The monthly mean precipitation varied from 25 to 225 mm in 2014 and 29 to 216 mm in 2015 during the giant ragweed growing season (Table S1). However, mean daily temperatures during the flowering period varied from 17 °C to 30 °C in 2014 and 20 °C to 30 °C in 2015 (Fig. 1). Average wind speed during the flowering period in 2014 and 2015 was 1.2 and 2.8 m s^−1^, respectively (Fig. 2). However, the pattern of wind flow was similar in both years, with wind blowing from the south (S) or southeast (SE) most of the time (Fig. 2). The correlation between PMGF and wind parameters (wind speed, wind frequency, or wind run) was inconsistent in magnitude and direction at different distances in both years (Tables S2 and S3). The interactions of distance × direction × year were significant (P < 0.05), suggesting that the frequency of gene flow varied between directions in each year and between years at specific distances.

### Flowering Synchrony

Initiation of flowering was observed on July 25 and July 30 in 2014 and 2015, respectively. The female flowers become receptive before the male flowers started shedding pollen. The protrusion of stigmas in the female flowers occurred on average 3 to 5 days prior to the pollen shed from male flowers on the same plant. GR giant ragweed plants in the center and GS plants within a 4 m distance from the pollen source in different directions flowered together, while flowering was delayed by 3 to 6 days in GS plants at distances ≥10 m from the pollen source. Peak flowering occurred 3 weeks after floral initiation, though continuous pollen production and a small number of new receptive stigmas were observed until mid-September. The total flowering period lasted 5 to 6 weeks with a flowering synchrony of ≥80% between GR and GS giant ragweed biotypes in both years (Table 1).

**Table 1:** Flowering synchrony between glyphosate-resistant and -susceptible giant ragweed in the pollen-mediated gene flow study conducted in 2014 and 2015 at Clay Center, Nebraska (NE), USA.

Directions	Flowering synchrony*							
	2014				2015			
	Aug 1	Aug 10	Aug 25	Sep 10	Aug 1	Aug 15	Aug 30	Sep 10
N	1.4	1.5	1.0	0.9	1.5	1.0	1.1	0.8
S	0.6	1.3	1.1	1.4	0.8	1.2	1.0	0.9
E	0.9	1.3	1.0	1.6	1.7	1.0	1.0	1.0
W	1.5	1.2	1.0	1.2	1.3	1.0	1.1	1.5
NE	1.1	1.5	1.0	1.0	1.3	1.0	1.0	0.9
NW	1.4	1.5	1.0	1.0	1.6	1.1	1.1	1.1
SE	1.3	1.4	1.0	1.0	1.6	1.0	1.2	1.2
SW	1.5	1.4	1.0	1.0	1.4	1.3	1.0	1.0
Average	1.2	1.4	1.0	1.1	1.4	1.2	1.1	1.1
% flowering plants in pollen-donor block	25	45	99	65	35	60	99	60

*Flowering synchrony between glyphosate-resistant and -susceptible giant ragweed was calculated using Equation: ${X}_{i}\,=\frac{1}{n}{\sum }_{j=1}^{n}\frac{A \% }{{B}_{j} \% }$, where *n* is the total number of distances in direction *i*, *A*% is the percentage of plants shedding pollen in the pollen-donor area, and *B*_*j*_% is the percentage of flowering plants at the *j* ^*th*^ observation (distance) in the pollen-receptor blocks at that specific time. *X* = 1.0 means perfect synchrony between the pollen donor and the receptor. *X* > 1.0 shows that sufficient pollens from GR male plants were present to pollinate GS females, but *X* values as low as 0.5 were not considered a good synchrony.

### Frequency of Gene Flow

**Figure 3:**
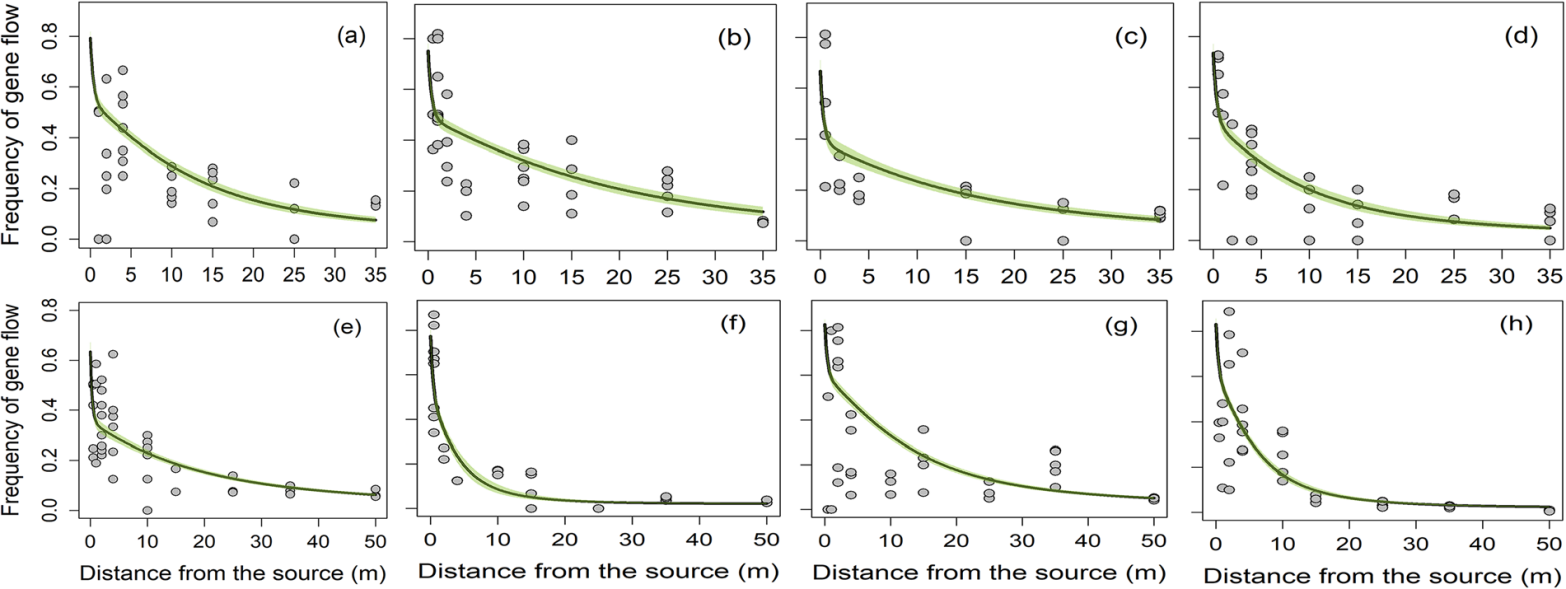
Pollen-mediated gene flow from glyphosate-resistant to -susceptible giant ragweed affected by distance (m) from the pollen source in eight directions: (**a**) East, (**b**) West, (**c**) North, (**d**) South, (**e**) Northeast, (**f**) Northwest, (**g**) Southeast, and (**h**) Southwest in 2014. The green shaded area represents the 95% confidence intervals for prediction plots.

**Figure 4:**
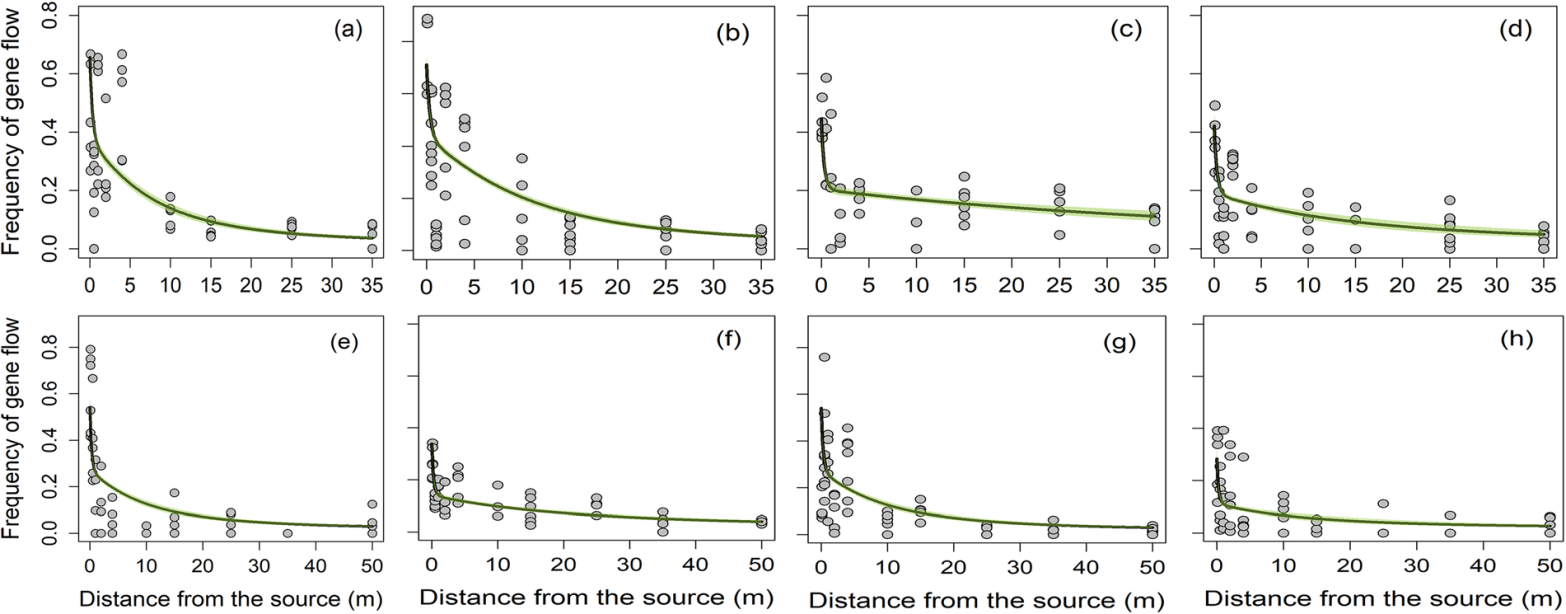
Pollen-mediated gene flow from glyphosate-resistant to -susceptible giant ragweed affected by distance (m) from the pollen source in eight directions: (**a**) East, (**b**) West, (**c**) North, (**d**) South, (**e**) Northeast, (**f**) Northwest, (**g**) Southeast, and (**h**) Southwest in 2015. The green shaded area represents the 95% confidence intervals for prediction plots.

A total of 100,938 giant ragweed plants were screened in the greenhouse and 16,813 plants were found resistant to glyphosate (Table 2). The frequency of gene flow declined with increasing distances from the pollen source following a leptokurtic pattern, though the magnitude varied between directions and years (Figs 3 and 4; Table 2). The highest frequency of gene flow averaged over eight directions was 0.54 to 0.60 (i.e., 54 to 60%) at ≤1 m distance in 2014 compared to 0.43 (43%) at the 0.1 m distance from the edge of the pollen-donor block in 2015 (Table 2). The average frequency of gene flow declined to ≤0.09 and ≤0.04 at the 35 and 50 m distances from the pollen source, respectively, in both years (Table 2).

**Table 2:** Pollen-mediated gene flow from glyphosate-resistant to -susceptible giant ragweed in a field experiment conducted in 2014 and 2015 at Clay Center, NE.

Distance from pollen-source	Plants screened*	Plants with glyphosate resistance trait	Frequency of gene flow^†^	Power, (1 − β)^‡^, α = 0.05
m	#	#		
**Year 2014** ^**╤**^				
0.5	2,591	1,546	0.60	>0.95
1	2,157	1,154	0.54	>0.95
2	3,201	1,281	0.40	>0.95
4	2,456	781	0.32	>0.95
10	2,325	498	0.21	>0.95
15	2,182	282	0.13	0.88
25	3,385	315	0.09	0.95
35	5,585	519	0.09	0.95
50	23,820	941	0.04	0.90
Total	47,702	7,317		
**Year 2015**				
0.1	5,198	2,218	0.43	>0.95
0.5	5,941	1,645	0.28	>0.95
1	5,247	1,120	0.21	>0.95
2	6,696	1,535	0.23	>0.95
4	6,376	1,488	0.23	>0.95
10	2,003	247	0.12	>0.95
15	4,782	437	0.10	0.88
25	3,661	278	0.08	0.95
35	3,285	202	0.06	0.80
50	10,047	326	0.03	0.92
Total	53,236	9,496		

^*^Total number of giant ragweed plants screened from all the eight directions at a specific distance from the pollen source. ^†^Average pollen-mediated gene flow frequency from all eight directions. Frequency of gene flow was determined using the equation, $Frequency\,of\,gene\,flow=\frac{Number\,of\,surviving\,plants}{Number\,of\,sprayed\,plants\,}$.

^‡^Value of power was calculated from a 95% confidence interval using the procedure described by Jhala *et al*. (2011). ^₸^The data for 0.1 m distance from 2014 was not included in the analysis because a sufficient sample size for a power of ≥0.8 was not available.

A double exponential decay model (Eq. 4) with distance, direction, and interaction of directions with year was selected as the best model out of 43 candidate models based on Akaike’s Information Criterion (*AIC*) (Table S4). All of the top competitive models suggested that inclusion of wind direction as a covariate in modeling was more appropriate compared to the inclusion of hourly wind data (wind speed, wind frequency, or wind run) (Table S4). The exponential decay curves indicated that the frequency of gene flow varied in different directions in both years (Figs 3 and 4). Furthermore, the estimated coefficients for the first or second order intercepts and decay rates were variable indicating that wind direction had a strong impact on the magnitude of the gene flow (Table 3). Irrespective of other factors, the highest collective PMGF in 2014 occurred in southeast (0.33) and west (0.33) directions followed by east direction (0.31) and in east (0.24) and west (0.23) direction in 2015 (Fig. S1).

**Table 3:** Estimation of the coefficients, standard error, and test of significance for the double-exponential decay model^*^ for the prediction of gene flow from glyphosate-resistant giant ragweed under field conditions.

Coefficients^†^	Estimate	Std. Error	z value	P-value^‡^
*β*_0_	−3.50	0.09	−39.57	<2.0e-16***
*β*_1_	0.26	0.10	2.56	0.0103*
*γ*_1_	−5.35	1.06	−5.04	4.74e-07***
*β*_2_	1.59	0.07	22.40	<2.0e-16***
*γ*_2_	−0.03	0.01	−2.65	0.0081**
*β*_2_: Direction N	−0.25	0.12	−2.19	0.0281*
*β*_2_: Direction NE	−0.59	0.09	−6.10	1.04e-09***
*β*_2_: Direction NW	1.04	0.10	10.20	<2.0e-16***
*β*_2_: Direction S	0.09	0.11	0.78	0.4343
*β*_2_: Direction SE	−0.12	0.12	−0.98	0.3260
*β*_2_: Direction SW	1.21	0.10	11.52	<2.0e-16***
*β*_2_: Direction W	−0.17	0.08	−2.18	0.0293*
*γ*_2_: Direction N	−0.02	0.02	−0.69	0.4877
*γ*_2_: Direction NE	0.24	0.04	6.18	6.35e-10***
*γ*_2_: Direction NW	−0.52	0.06	−9.19	<2.0e-16***
*γ*_2_: Direction S	−0.02	0.02	−0.80	0.4235
*γ*_2_: Direction SE	−0.02	0.02	−1.25	0.2080
*γ*_2_: Direction SW	−0.05	0.03	−1.66	0.0955
*γ*_2_: Direction W	0.02	0.01	2.09	0.0361*
*β*_2_: Year 2	−0.23	0.04	-6.10	1.04e-09***
*γ*_2_: Year 2	−0.02	0.01	−2.41	0.0156*
*β*_2_: Direction N:Year 2	−0.04	0.06	−0.66	0.5074
*β*_2_: Direction NE:Year 2	0.31	0.05	5.45	5.04e-08***
*β*_2_: Direction NW:Year 2	−0.78	0.06	−12.81	<2.0e-16***
*β*_2_: Direction S:Year 2	−0.23	0.06	−3.66	0.0002***
*β*_2_: Direction SE:Year 2	−0.02	0.06	−0.31	0.7520
*β*_2_: Direction SW:Year 2	−0.94	0.07	−13.12	<2.0e-16***
*β*_2_: Direction W:Year 2	0.12	0.04	2.89	0.0038**
*γ*_2_: Direction N:Year 2	0.03	0.00	3.32	0.0008***
*γ*_2_: Direction NE:Year 2	−0.23	0.03	−6.29	3.13e-10***
*γ*_2_: Direction NW:Year 2	0.27	0.03	9.44	<2.0e-16***
*γ*_2_: Direction S:Year 2	0.02	0.01	1.68	0.0912
*γ*_2_: Direction SE:Year 2	0.02	0.01	2.07	0.0383*
*γ*_2_: Direction SW:Year 2	0.01	0.02	0.53	0.5926
*γ*_2_: Direction W:Year 2	−0.01	0.00	−0.49	0.6238

*[*logit*(*p*_*i*_) = *β*_0_ + exp[*β*_1_ + *γ*_1_ × *Distance*] + exp[*β*_2_(*Direction*:*Year*) + *γ*_2_(*Direction*:*Year*) × *Distance*], where *p*_*i*_ is frequency of gene flow of the *i* ^*th*^ observation; *β*_0_ is the overall intercept; *β*_1_ and *β*_2_ are the intercepts for the first and second instances, respectively; and *γ*_1_, and *γ*_2_ are the decay rates. ^†^ *β*_2_ and *γ*_2_ vary with the direction and the year. In this table, *β*_2_ and *γ*_2_ show the intercept and decay rate, respectively, for one direction (East) in year 1 (2014). However, “*β*_2_:Direction”, or “*β*_2_:Year 2” denote the change (from East direction and year 1) in *β*_2_ for other directions and year 2 (2015), respectively. The same is true for *γ*_2_. ^‡^P-values show the test of significance at P < 0.05 (*) and P < 0.01 (**).

The predicted distances where gene flow was reduced by 50% (*O*_*50*_) varied from 1.3 m to 7.0 m in 2014; and from 0.3 m to 2.4 m in 2015 (Table 4), depending on the direction. Similarly, the predicted distances for 90% (*O*_*90*_) reduction in gene flow varied depending on the direction. For example, the maximum distance at which 90% reduction in gene flow occurred was 49.5 m in the W arm in 2014 and 106.5 m in the N arm in 2015. Large confidence intervals of the predicted distances at which 90% reduction in gene flow occurred suggested a higher variability in frequency of gene flow at further distances from the pollen source (Table 4). In addition, some of the predicted *O*_*90*_ values are greater than the maximum distance (50 m) measured in this study making it difficult to maintain the accuracy achieved at the closer distances (Table 4).

**Table 4:** Estimates of the distances where the frequency of gene flow reduced by 50% (*O*_*50*_) and 90% (*O*_*90*_) in 2014 and 2015 and their respective confidence intervals from logistic regression analysis^*^.

Direction	2014				2015			
	*O*_*50*_	*CI*	*O*_*90*_	*CI*	*O*_*50*_	*CI*	*O*_*90*_	*CI*
**m**								
N	1.3	0.4; 4.1	45.6	32.1; 64.4	0.4	0.3; 0.5	106.5	79.3; 142.2
S	2.8	0.8; 4.3	28.6	20.5; 40.1	0.4	0.3; 0.4	37.1	27.3; 49.6
E	4.5	3.4; 5.4	27	22.7; 32.2	1.1	0.8; 1.5	19	16.6; 21.6
W	7.0	5.8; 8.3	49.5	42.7; 57.5	2.4	2; 2.8	26.4	24; 29.1
NE	1.3	0.5; 2.6	35.1	25.4; 48.3	0.5	0.4; 0.6	4.4	3.6; 5.4
NW	1.4	1.3; 1.6	4.9	4.2; 5.6	0.3	0.3; 0.4	46.9	34.5; 63.5
SE	2.5	0.7; 3.9	25.5	20.2; 32.3	0.6	0.4; 0.7	29.4	24.5; 35.3
SW	5.1	4.7; 5.4	17.5	16.1; 19.0	0.3	0.2; 0.3	26	13.6; 53.6

*O_*50*_ and *O*_*90*_ are the predicted distances for 50% and 90% reduction in gene flow; *CI* is the 95% confidence interval, which includes the lower and upper limits. *O*_*50*_ and *O*_*90*_ were determined from the final model [*logit*(*p*_*i*_) = *β*_0_ + exp[*β*_1_ + *γ*_1_ × *Distance*] + exp[*β*_2_(*Direction*:*Year*) + *γ*_2_(*Direction*:*Year*) × *Distance*] using the prediction function in R.

## Discussion

The results of this study indicated protogynous nature and extended flowering period (from mid-July to late August or early September) in the giant ragweed and were in consensus with the earlier reports by Bassett and Crompton^52^. The extended flowering period increases synchrony among flowering plants and protogyny favors gene flow^55,56^. The delayed flowering at distances ≥10 m from the pollen source was possibly due to lower plant density and minimal competition for resources resulting in vigorous vegetative growth and a delay in the transition to the reproductive phase, however, flowering synchrony was not affected.

A double exponential decay model was used to describe PMGF in this study. Sarangi *et al*.^45^ and Bagavathiannan and Norsworthy^42^ used a similar approach to determine PMGF in common waterhemp (*Amaranthus rudis* Sauer) and barnyardgrass [(*Echinochloa crus-galli* (L.) Beauv.], respectively. Furthermore, the correlation analysis and model selection procedure indicated wind direction as a more relevant predictor of PMGF compared to wind parameters, including wind speed, wind frequency, or wind run. High temporal variation in wind frequency or wind run and wind gusts may be the reason for the absence of a strong correlation between wind parameters and the frequency of PMGF. Because giant ragweed is a monoecious species, pollen competition is expected between GR and locally available GS pollen from the pollen receptor plants for successful events of gene flow, reducing the chances of gene flow through GR pollen, primarily carried by wind. While historically, very few PMGF studies have included the effect of wind direction in quantifying the frequency of gene flow^45,57,58^, wind direction should be included to reduce the potential of the over- or under estimation of gene flow. Recently, Beckie *et al*.^59^ reported that PMGF from GR to GS kochia [*Kochia scoparia* (L.) Schrad] was influenced by wind direction; however, PMGF in common lambsquarters (*Chenopodium album* L.) did not depend on wind direction^48^.

PMGF reported in this study is relatively greater than the 31% reported by Brabham *et al*.^50^ between GR and GS giant ragweed planted in rows side-by-side at a distance of 0.76 m. This might be because GR pollen source was only in one direction and from limited number of plants, while our study had several GR giant ragweed plants contributing to create a pollen cloud. Raynor *et al*.^60^ reported that approximately 9% of the ragweed (*Ambrosia*) pollen released from the pollen source reached up to a distance of 60 m. A relatively high level of gene flow in giant ragweed compared to other self-compatible species such as common lambsquarters^48^ and giant foxtail (*Setaria faberi* Herrm.)^61^ is likely due to its facultative outcrossing nature, as favored by anemophilous pollination, ability of massive pollen production^51^ and absence of any physical barriers. Several studies have documented that PMGF has a significant role in transferring and altering the frequency of resistant alleles within and between weed populations; for example, in a predominantly self-pollinated weed species such as common lambsquarters, PMGF varied from 3% at 2 m to 0.16% at 15 m from the pollen source to the receptor biotype^48^. Similarly, PMGF in giant foxtail ranged from 0.24% and 0.73% among plants grown 0.36 m apart^61^. Recently, Beckie *et al*.^59^ reported 5.3 to 7.5% gene flow at ≤1 m distance from GR to GS kochia, which declined exponentially to 0.1 to 0.4% at the 96 m distance. In contrast, gene flow from imidazolinone-resistant domesticated sunflower (*Helianthus annuus* L.), a cross-pollinated species, to wild sunflower ranged from 11 to 22% and 0.3 to 5% at 2.5 and 30 m, respectively, from the pollen source^62^.

The results of this study suggested that PMGF plays a significant role in the dispersal of GR alleles in giant ragweed, causing an increase in the frequency of GR giant ragweed plants within field populations along with the potential to introduce GR alleles into nearby field or non-crop giant ragweed populations. Similarly, Sarangi *et al*.^45^ reported PMGF from GR common waterhemp to GS common waterhemp and the potential spread of resistance alleles through pollen. It has been reported that giant ragweed is known to grow on field edges, in fallow, or other non-crop situations^51,52^; therefore, it is possible that herbicide-resistant alleles can be transferred through PMGF to isolated giant ragweed populations under non-crop situations. In addition to gene flow, the dynamics of resistance in a population are determined by the initial frequency of the resistant alleles, along with their heritability, reproduction, and fitness^34,49^. Maxwell *et al*.^63^ identified two sets of biological processes that influence ecological fitness and gene flow as key factors in the evolution and dynamics of herbicide-resistant weed populations. Studies on the relative fitness of GR and GS giant ragweed reported contrasting results^50,64^. Brabham *et al*.^50^ reported that the fitness penalty in a GR giant ragweed biotype from Indiana resulted in low fecundity in GR plants compared to GS plants, though the authors mentioned that a different origin of the two biotypes might be the reason for differences in fecundity. In contrast, Glittner and Stoltenberg^64^ reported more fecundity and similar viability in the GR giant ragweed biotype compared to a GS biotype from Wisconsin in the absence of glyphosate. In the absence of a fitness penalty, GR plants with greater fecundity will likely contribute higher proportions of GR seeds into the soil seedbank, leading to an increased number of plants with the GR trait in the giant ragweed population even in the absence of glyphosate^64^. Therefore, a high frequency of PMGF and the lack of fitness penalty in GR giant ragweed makes it ideal for spreading the glyphosate resistance trait. A recent survey reported that herbicide-resistant giant ragweed is widespread in the eastern Corn Belt of the United States and did not remain confined to sites where it first evolved^65^. It is possible that its widespread occurrence is not only due to seed movement, and that pollen movement may have contributed significantly.

This is the first report of the long-distance dispersal of GR alleles in giant ragweed under field conditions. The results of this study are critical to explaining the widespread occurrence of GR giant ragweed in the Midwest and may be useful in developing a simulation model to predict the spread of resistant alleles or the dissemination of multiple herbicide resistance alleles from their point of origin. PMGF enhances genetic variance in a population and increases the frequency of multiple or polygenic herbicide resistance and the evolutionary dynamics of a species^7,66^. For example, two distinct GR phenotypes— the rapid necrosis response and slow response biotypes—have been reported in giant ragweed^50,67^, supporting the possibility that different mechanisms of resistance are involved. Though the precise mechanism(s) of herbicide resistance in giant ragweed is unknown^67^, the partial role of altered translocation has been suggested^68,69^. It is possible that PMGF may bring rapid and slow response mechanisms together and result in the evolution of GR populations with more complex mechanism(s) of resistance. Similarly, it is also possible that giant ragweed biotypes resistant to ALS-inhibitors and glyphosate might have evolved due to PMGF, and more such cases should be expected in the future due to the widespread occurrence of ALS- and glyphosate-resistant giant ragweed in the Midwestern United States. In a recent survey, Regnier *et al*.^65^ also reported that herbicide resistance to ALS-inhibitors and glyphosate in giant ragweed were concentrated in the same counties and clusters of counties with multiple modes of resistance in Ohio, Indiana, Illinois, Missouri, Iowa, Nebraska, and Minnesota. The same study reported that out of 15 states surveyed, resistance to ALS-inhibitors, glyphosate, and both (ALS + glyphosate) modes of action occurred in 13, 14, and 12 states in contrast to confirmed reports from 5, 12, and 3 states, respectively.

For pollen to be effective in fertilizing over long-distance gene dispersal, extended pollen viability is required^70^. The characteristics of giant ragweed pollen, including its nearly spherical shape, the presence of numerous spine-like projections on its surface, its small pollen size varying from 18 to 25 µm, and its low velocity of deposition (0.02 to 0.06 m s^−1^), likely favor long-distance pollen dispersal^71–73^. However, scientific literature on the duration of pollen viability in giant ragweed is not known. Additionally, since this study was conducted under non-crop conditions with a small pollen source relative to the natural stands of GR giant ragweed under field conditions, the results may vary compared to field conditions with crops or other weed species acting as vegetation barriers that result in a different ratio of GR to GS plants. Therefore, future studies should consider evaluating the duration of pollen viability and the landscape-level dissemination of the GR trait in giant ragweed.

### Practical Implications of PMGF in Glyphosate-Resistant Giant Ragweed Management

Based on the results of this study, it is evident that pollen-mediated dissemination of the GR trait is possible in giant ragweed and depends on multiple factors, including distance from the pollen source, wind speed, and wind direction. Therefore, necessary adjustments in management approach are needed, including the control of giant ragweed escapes before flowering and communication and collaboration among growers to avoid the farm-to-farm spread of herbicide resistance. Further awareness among growers about the significance of PMGF in the spread of resistance genes from herbicide-resistant to -susceptible weed species is needed^41,42^. The adoption of integrated weed management approaches with diversified strategies should be encouraged to avoid the widespread dispersal of existing herbicide resistance traits as well as to delay the evolution of new herbicide-resistant weeds^74,75^, and mitigate transgene flow^39^.

## Materials and Methods

### Plant Material

Seed heads of the GR giant ragweed biotype were collected in 2013 from a grower’s field near David City, Nebraska (NE) (41.26 °N, 97.14 °W). The level of glyphosate resistance in this biotype was 14-fold compared to a known susceptible biotype^76^. Similarly, seed heads were collected from a known GS giant ragweed biotype from the South Central Agricultural Laboratory (SCAL), University of Nebraska-Lincoln near Clay Center, NE (40.58 °N, 98.14 °W). Seed heads were manually threshed using a handheld roller and cleaned using a seed blower (South Dakota Seed Blower, Seedburo Equipment Co., 1022 W. Jackson Blvd., Chicago, IL). To overcome dormancy, giant ragweed seeds were packed in mesh bags, placed between moistened layers of soil in plastic boxes, and stored in a freezer at −8 °C for 3.5 months for use in this study^77^.

Seeds from the GR and GS biotypes were germinated in 72-celled plastic germination trays containing potting mix (Berger BM1 All-Purpose Mix, Berger Peat Moss Ltd., Saint-Modeste, Quebec, Canada). One plant per cell was maintained after two weeks and extra plants were transplanted to additional germination trays to raise vigorous seedlings for transplanting. Plants were maintained in the greenhouse with a daytime temperature of 25 ± 2 °C, a nighttime temperature of 18 ± 3 °C, and a relative humidity of 70 to 75%. Sodium halide lamps were used as a supplemental light source to ensure a 15-h photoperiod. Plants were supplied with adequate nutrients [0.075% w/v solution of Miracle-Gro Water Soluble All Purpose Plant Food (N-P_2_O_5_-K_2_O:24-8-16), Scotts Miracle-Gro Products Inc., 14111 Scottslawn Road, Marysville, OH 43041] every two weeks and watered daily, except during the week before transplanting when water was added alternately to acclimatize the plants. Glyphosate response of the GR and GS biotypes was further verified in both years by treating a randomly selected sample of 100 plants from each biotype with 1 × (1,260 g ae ha^−1^) rate of glyphosate (Fig. S2); however, no anomalous plants were observed. The seedlings of both biotypes were then transplanted to the field when the majority of plants had attained an 8 to 12 cm height.

### Field Experiments

A field experiment was conducted in 2014 and 2015 at South Central Agricultural Laboratory (SCAL), University of Nebraska-Lincoln at Clay Center. The soil texture at the experimental site was Crete silt loam (fine, montmorillonitic, mesic, Pachic Argiustolls) consisting of 17% sand, 58% silt, 25% clay, 2.5% organic matter, and a pH of 6.5. The primary weed species observed at the experimental site were common lambsquarters, common waterhemp, green foxtail [*Setaria viridis* (L.) Beauv.], Palmer amaranth (*Amaranthus palmeri* S. Wats.), and velvetleaf (*Abutilon theophrasti* Medik.). There was no suspicion or report of any GR weed species on or around the experimental site. Field preparation began in early May with tillage using a tandem disk harrow followed by an application of micro-encapsulated acetochlor (1.68 kg ai ha^−1^) (Warrant^®^, Monsanto Company, 800 N, Lindbergh Blvd., St. Louis, MO 63167) tank-mixed with glyphosate (0.87 kg ae ha^−1^) (Roundup PowerMax^®^, Monsanto Company, 800 N, Lindbergh Blvd., St. Louis, MO 63167) to control early-season weeds. Later in the season, the experimental site and its surrounding area (up to 60 m) was kept weed-free either by hand-weeding or cultivation. The experiments were conducted under non-crop conditions without any physical barriers to obstruct natural wind or pollen movement.

**Figure 5:**
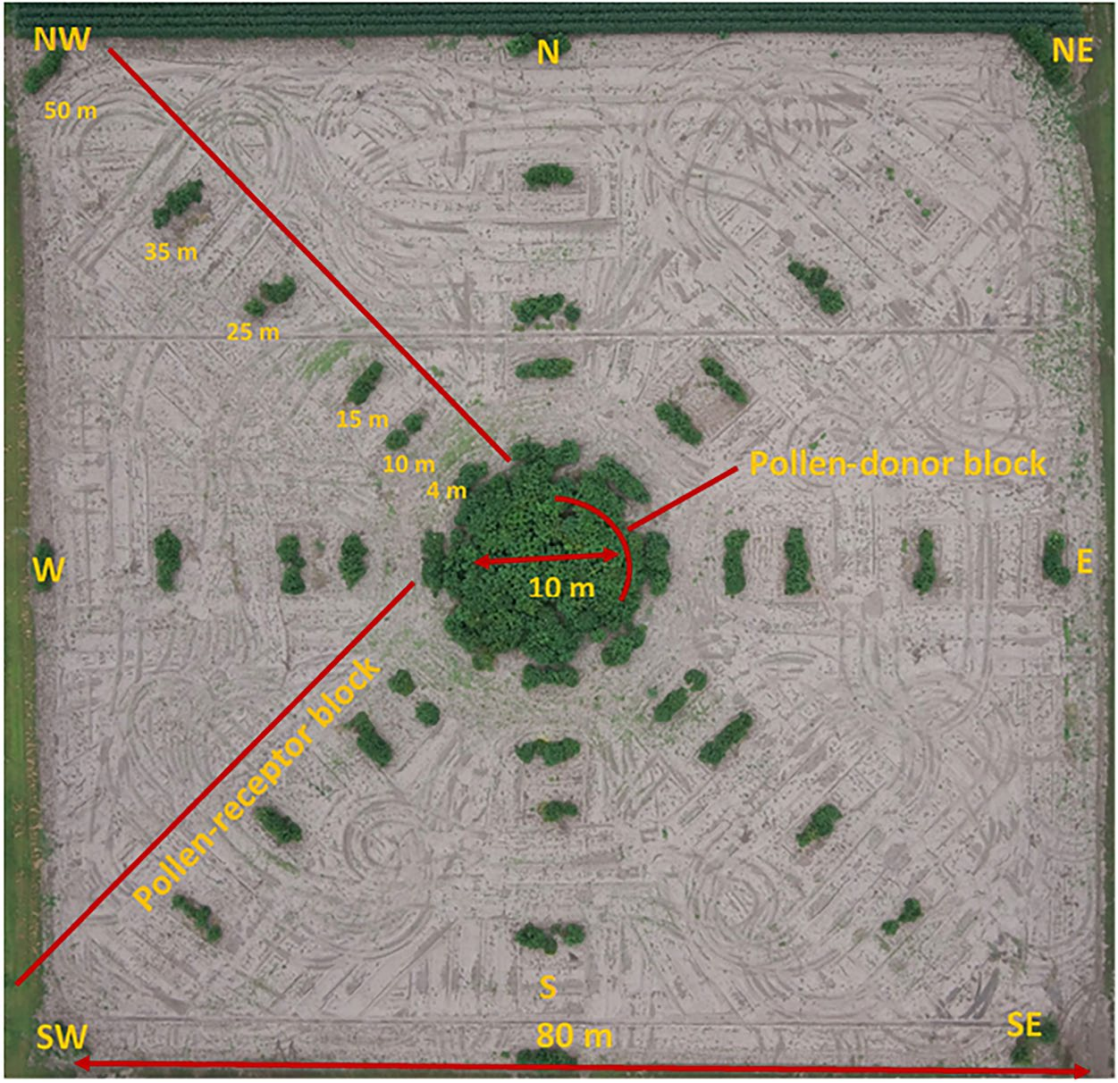
Aerial view of the field experiment conducted to quantify pollen-mediated gene flow from glyphosate-resistant to -susceptible giant ragweed at South Central Agricultural Laboratory (SCAL), Clay Center, Nebraska, USA. Glyphosate-resistant giant ragweed plants were transplanted in the pollen-donor block of 10 m diam in the center of the field. The surrounding pollen-receptor area (80 m × 80 m) was divided into eight directional blocks where glyphosate-susceptible giant ragweed plants were transplanted. Giant ragweed seeds were harvested at maturity from specific distances along the eight directional arms. Aerial image is courtesy of Dr. Richard Ferguson, University of Nebraska-Lincoln.

Field experiments were conducted using a modified Nelder wheel design^17,78,79^ with the pollen source (GR giant ragweed) planted in the center and the pollen receptors (GS giant ragweed) planted around the center. The experimental area was 80 m × 80 m with a central circle of 80 sq m (10 m diameter) for the pollen-donor block (Fig. 5). Each year about 377 GR giant ragweed plants were transplanted in the pollen donor block in East-West and North-South directions in a grid pattern with a 0.46 m plant to plant distance. The transplanting was performed on June 9 in 2014 and May 26 in 2015.

The receptor area was divided into eight directional blocks (cardinal: N, S, E, and W; and ordinal: NE, NW, SE, and SW) and six plants of the GS biotype were transplanted with a plant to plant spacing of 0.3 m at each of the specified distances (0.1, 0.5, 1, 2, 4, 10, 15, 25, 35 m for all cardinal and ordinal directions; and an additional 50 m only for the ordinal directions) from the pollen-donor block (Fig. 5).

### Meteorological Data

Hourly surface meteorological data including air temperature, precipitation, relative humidity, wind speed, and wind direction were recorded by installing a weather station (Onset Computer Corporation, 470 MacArthur Blvd., Bourne, MA 02532) at the experimental site. Wind frequency (frequency of time during which the wind blows in a certain direction), wind speed, and wind run (calculated by multiplying the average wind speed by the wind frequency)^11^ data were used for modeling PMGF, while other meteorological data such as temperature, humidity, and precipitation were also recorded due to their effect on pollen viability and dispersal^80^.

### Flowering Period and Seed Harvesting

The percentage of flowering plants was noted at 5 d intervals for the pollen-donor and -receptor blocks, and flowering synchrony was evaluated for each direction using the equation^45^:

1 $$Flowering\,synchrony\,( \% )=\frac{1}{n}{\sum }_{i}^{n}\frac{({A}_{i} \% )}{(B \% )}\times 100$$

where *n* is the total number of distances in each direction, *A*_*i*_% is the percentage of flowering plants at the *ith* observation (distance) in the pollen-receptor blocks, and *B*% is the percentage of plants shedding pollen in the pollen-donor area at that particular time. *A* ≥ *B* indicates fully synchronized flowering (i.e., 100%) in the pollen receptor.

At maturity, the seed heads of the GS giant ragweed plants from each distance and direction were hand-harvested, bagged, and separately labeled. The harvested seeds were cleaned thoroughly, and stratified to break seed dormancy using the same procedure described previously.

### Resistance Screening

Greenhouse dose-response bioassays for the parent biotypes (GR and GS) were conducted and the effective doses of glyphosate required for 50% (ED_50_) and 90% (ED_90_) injury of the parent biotypes were determined using the *drc* package in R software (R statistical software, R Foundation for Statistical Computing, Vienna, Austria)^81^. Control was visually estimated and recorded at 21 d after treatment using 0% to 100% scale with 0% meaning no control or injury and 100% meaning complete mortality of the treated plant. Percent control of treated plants was assessed based on comparison with the nontreated control plants with respect to symptoms such as chlorosis, necrosis, stand loss, and stunting. The ED_50_ values for the GR and GS biotypes were 1,722 and 112 g ae ha^−1^, respectively, whereas the ED_90_ values were 14,254 and 468 g ae ha^−1^, respectively (Fig. S3).

Seeds collected from the GS giant ragweed plants were germinated separately for each distance and direction in the greenhouse and evaluated for glyphosate resistance. Plastic trays (51 cm × 38 cm × 10 cm) containing potting mix (described previously) were used for growing the plants. A maximum of 130 plants were allowed per tray to ensure sufficient glyphosate coverage on the leaf surface. The plants were sprayed at an 8–10 cm height with 2 × the recommended rate of glyphosate (Touchdown HiTech^®^, Syngenta Crop Protection, LLC, P.O. Box 18300, Greensboro, NC 27419–8300), where 1 × =1,260 g ae ha^−1^. The resistance screening was performed at the 2× rate (2,520 g ae ha^−1^) of glyphosate to obtain more consistency in the glyphosate response of the giant ragweed plants with complete mortality of all susceptible plants present, and to assure the survival of any GR plant (as the ED_90_ value for the GR parent plants was 5.6-times higher than the 2× rate of glyphosate; Fig. S3). The number of seedlings surviving glyphosate treatment were recorded at 21 d after application and the frequency of gene flow at each distance/direction was calculated using the equation:

2 $$Frequency\,of\,gene\,flow=\frac{Number\,of\,surviving\,plants}{Number\,of\,plants\,screened}$$

### Sampling Strategy

A sampling strategy suggested by Jhala *et al*.^17^ was followed to select a sample size for screening giant ragweed plants to quantify the PMGF with a power of ≥0.8. Power analysis with binomial probabilities was used to determine the minimum sample size needed to accept the results of a statistical test with a particular level of confidence. Following the procedure of Jhala *et al*.^17^ minimum sample sizes were estimated for different theoretical frequencies at three different confidence intervals (α) and power values (1−β). The observed frequencies from the study were then compared with the theoretical frequencies, and the gene flow was considered significant if the observed frequencies were greater than the theoretical frequencies.

### Statistical Analysis

An information-theoretic approach^82–84^ of the model selection was used to select the best model for analyzing the PMGF between GR and GS giant ragweed. Unlike traditional null hypothesis testing, the model selection approach allows simultaneous evaluation of multiple competing hypotheses (models) rather than only two hypotheses (the null and a single alternative hypothesis)^84,85^.

Frequency of gene flow usually follows a binomial distribution, and the two possible outcomes in this study were either dead (susceptible) or live (resistant) giant ragweed seedlings after screening with glyphosate. A characteristic of binomial distribution is that mean and variance are equal and dependent on the underlying probability function, p_i_. A set of 43 possible models were constructed to explain the frequency of PMGF using an exponential decay function with distance from the pollen source, direction of the pollen-receptor blocks, average wind speed, wind frequency, and/or wind run as the explanatory variables in different logically possible combinations without collinearity. The nonlinear regression models were fit using the Generalized Nonlinear Models (gnm) package in R software. The advantage of using the gnm compared to the nonlinear least square (*nls*) function is that responses with non-Gaussian distribution can be fitted, in addition to their convenience in representing a model with a large number of parameters through symbolic model specification^86^. Similarly, correlation analysis between PMGF and wind parameters was conducted in R software using *cor* and *cor.test* functions.

### Model Selection

The Akaike’s Information Criterion (*AIC*) was followed to compare the candidate models and select the best model using the equation^87^:

3 $$AIC=-2LL+2K$$

where *LL* is the log-likelihood function for the models and *K* is the number of parameters estimated. The lower the *AIC* value, the better the model; therefore, the model with the lowest *AIC* value was considered the best candidate model^88^.

### Best Model

The best fit to the data was provided by a double exponential decay model (Equation 4; Table 4) where the frequency of the PMGF varied with the distance from the pollen source, the direction of the pollen-receptors, and the year:

4 $$\begin{array}{rcl}logit({p}_{i}) & = & {\beta }_{0}+\exp [{\beta }_{1}+{\gamma }_{1}\times distance]+\exp [{\beta }_{2}(direction:year)\\  &  & +\,{\gamma }_{2}(direction:year)\times distance\end{array}$$

where *p*_*i*_ is the frequency of gene flow of the *i* ^*th*^ observation; *β*_0_ is the overall intercept; *β*_1_, *β*_2_ are the intercepts for the first and second instances, respectively; and *γ*_1_, *γ*_2_ are the decay rates where *γ*_1_ > *γ*_2_. Here, *β*_2_ and *γ*_2_ vary with the direction and the year.

In binomial distribution, probability (*p*_*i*_) is the function of the covariate (*γ*_*i*_ *x*_*i*_) (*x* is the distance from the pollen source) that can take any real value. Because the *p*_*i*_ ranges between 0 and 1 (0 ≤*p*_*i*_ ≤ 1), transformation of the probability becomes important in removing the range and floor restrictions. *Logit*, or log-odds were calculated using the transformation methods described by Cramer^89^, whereas the back-transformed data were presented:

5 $$\begin{array}{rcl}Logit,\,{\eta }_{i} & = & logit\,({p}_{i})=\,\mathrm{ln}(\frac{{p}_{i}}{1-{p}_{i}})\\ Back\,transformation,\,{p}_{i} & = & logi{t}^{-1}\,({\eta }_{i})=\frac{{e}^{{\eta }_{i}}}{1+{e}^{{\eta }_{i}}}\end{array}$$

The distances where the frequency of gene flow was reduced by 50% (*O*_*50*_) and 90% (*O*_*90*_) of the frequency predicted at the closest distance were estimated from the final model (Equation 4).

### Model Goodness of Fit

The goodness of fit statistic was estimated for the best model by measuring the difference between the observed and fitted values. Model goodness of fit was determined by *Pearson’s chi* − *squared statistic*, which can be written for binomial data as [Equation 6]

6 $${{\chi }^{2}}_{(n-k-1)}={\sum }_{i}\frac{{n}_{i}{({y}_{i}-{\hat{\mu }}_{i})}^{2}}{{\hat{\mu }}_{i}({n}_{i}-{\hat{\mu }}_{i})}$$

where the sum of the squared differences between *y*_*i*_ (observed values) and ${\hat{\mu }}_{i}$ (fitted values for the *i* ^*th*^ group of observations) was divided by the variance of *y*_*i*_ that was *μ*_*i*_(*n*_*i*_ − *μ*_*i*_)/*n*_*i*_ (with *μ*_1_ estimated using ${\hat{\mu }}_{i}$), and *n*_*i*_ is the sample size for the *i* ^*th*^ group. The degree of freedom for Pearson’s chi-squared statistic was *n* − *k* − 1, where *n* refers to the total number of groups and *k* refers to the number of parameters.

### Data availability

Raw data of this manuscript is available to download at https://unl.box.com/s/frkna57a9dncbqozly7zq17rogrnuao9.

### Significance

Glyphosate-resistant giant ragweed was first reported in Ohio, USA and has now been confirmed in 12 other states in the United States, including Nebraska. About 0.45 million hectares of corn and soybean fields in Nebraska are infested with glyphosate-resistant giant ragweed, in addition to several million hectares infested in other states of the Midwestern United States. It is an anemophilous facultative outcrossing species; therefore, evaluation of pollen-mediated gene flow (PMGF) and the role of wind are crucial for understanding the occurrence, and spread of this species. Modeling gene flow in giant ragweed provided useful information about the possibility of PMGF in disseminating resistance genes and the role of wind parameters.

## Electronic supplementary material

Supplementary Information
