# Supplementary material for: Modeling pollen-mediated gene flow from glyphosate-resistant to -susceptible giant ragweed (Ambrosia trifida L.) under field conditions
Source: Sci Rep. 2017 Dec 6;7:17067. doi: 10.1038/s41598-017-16737-z (PMC5719015; doi:10.1038/s41598-017-16737-z)
Supplement: Supplementary file 1 — Supplementary Information [file 41598_2017_16737_MOESM1_ESM.pdf]

Modeling pollen-mediated gene flow from glyphosate-resistant to -susceptible giant ragweed (*Ambrosia trifida* L.) under field conditions of co-existence

Zahoor A. Ganie[1], Amit J. Jhala

[1] Department of Agronomy and Horticulture, University of Nebraska–Lincoln, Lincoln 68583 Nebraska, USA.

Author contributions: Z.A.G, and A.J.J designed research; Z.A.G. performed research; A.J.J. contributed new reagents or analytic tools; Z.A.G. analyzed data; and Z.A.G. wrote the paper.
Short Title: Pollen-mediated gene flow in giant ragweed

The authors declare no conflict of interest.

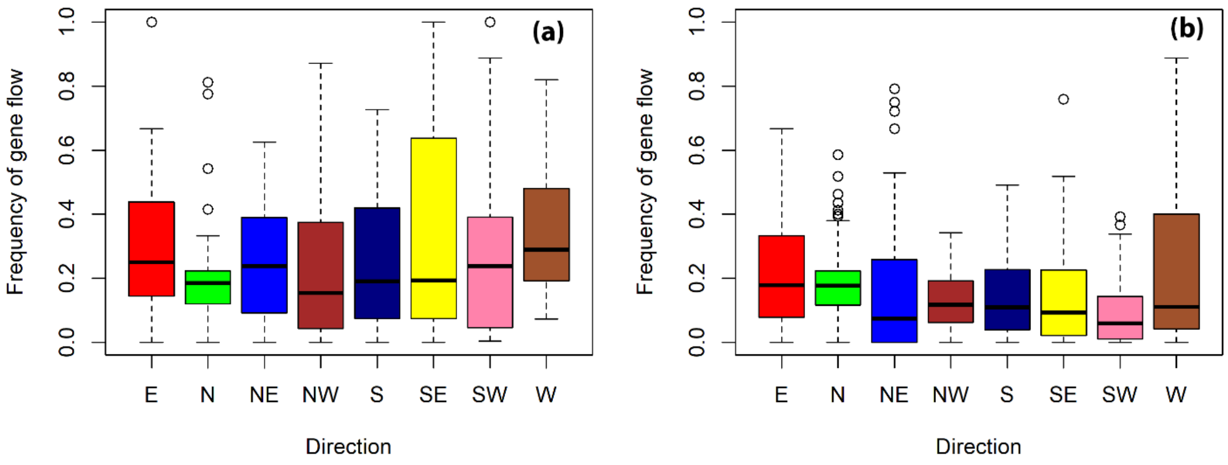

Fig. S1. Box-plots of frequency of gene flow over different direction irrespective of distance in 2014 (a) and 2015 (b).

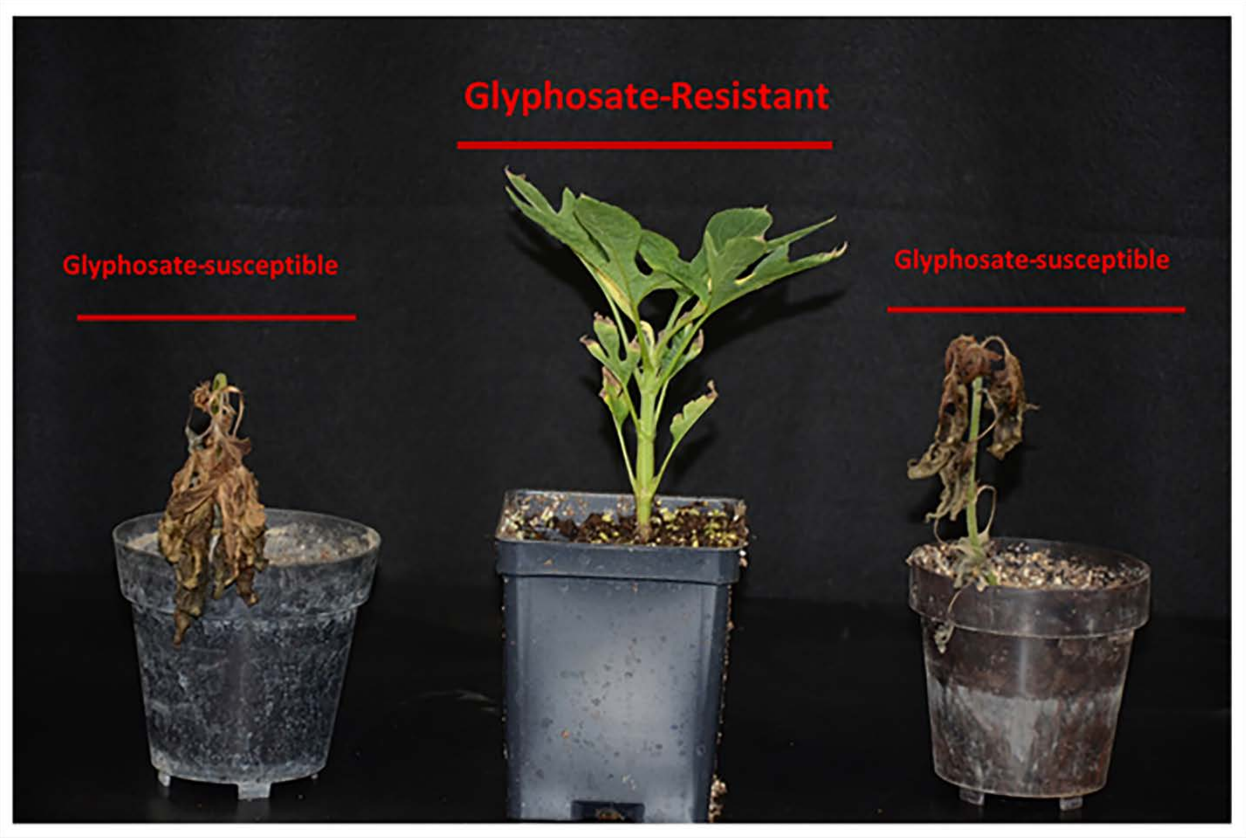

Fig. S2. Glyphosate-resistant and -susceptible giant ragweed plants at 14 d after treatment with glyphosate at 1× rate (1,260 g ha^{-1}) from the respective biotypes used as a pollen source and receptors in a gene flow study conducted in Nebraska.

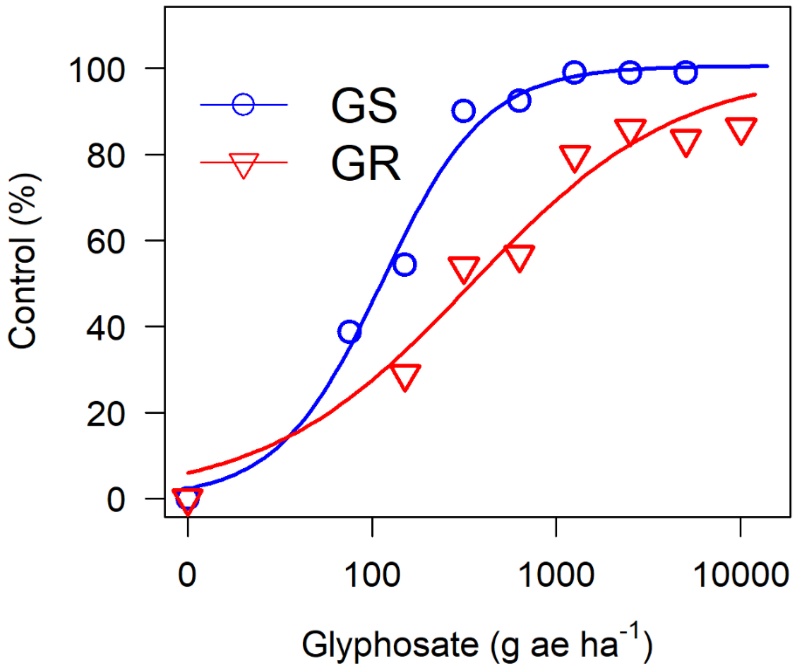

Fig. S3. Dose-response bioassay of glyphosate-resistant (GR) and -susceptible (GS) giant ragweed used as parent biotypes in this study. Giant ragweed control estimates were taken at 21 d after glyphosate application.

Table S1. Monthly mean air temperature and total precipitation during the 2014 and 2015 growing seasons and 30 year (yr) average at Clay Center, Nebraska, USA[a].

Month	Mean Temperature			Total Precipitation		
	2014	2015	30-yr average	2014	2015	30-yr average
	———C———			———mm———		
May	17	16	17	59	99	124
June	23	25	22	194	216	117
July	24	24	25	45	150	86
August	23	23	24	225	47	88
September	22	22	19	52	40	86
October	14	15	12	25	29	59
Annual	10	12	11	691	796	763

[a] Mean air temperature and total precipitation data were obtained from a field weather station and the 30-yr averages were obtained from the National Weather Service and Cooperative Observer Network (2016).

Table S2. Spearman correlation coefficients (r_s) between wind parameters (wind speed, wind frequency, and wind run) and frequency of gene flow at different distances in 2014*.

Wind parameters		Gene flow frequency	Distance from the pollen source†								
			0.5	1	2	4	10	15	25	35	50
			m								
Wind speed	r_s	- 0.082	0.21	0.37	0.04	0.06	- 0.40	0.02	0.02	0.09	0.08
	P-value	0.208	0.260	0.084	0.816	0.705	0.011	0.910	0.922	0.533	0.825
Wind frequency	r_s	- 0.077	0.02	0.19	- 0.11	- 0.22	- 0.52	0.29	- 0.09	- 0.02	0.72
	P-value	0.176	0.915	0.389	0.503	0.165	0.002	0.136	0.636	0.875	0.013
Wind run	r_s	- 0.112	0.17	0.18	- 0.06	- 0.08	- 0.49	0.13	- 0.07	0.10	0.41
	P-value	0.066	0.355	0.389	0.739	0.59	0.005	0.523	0.693	0.490	0.213

* Spearman correlation coefficients (r_s) were tested at a significance level of $P < 0.05$.

† Gene flow at 50 m was from the four ordinal directions (NE, NW, SE, SW) in 2014.

Table S3. Spearman correlation coefficients (r_s) between wind parameters (wind speed, wind frequency, and wind run) and frequency of gene flow at different distances in 2015*.

Wind parameters		Gene flow frequency	Distance from the pollen source†									
			0.1	0.5	1	2	4	10	15	25	35	50
			m									
Wind speed	r_s	- 0.04	- 0.10	- 0.06	0.10	- 0.09	- 0.06	- 0.14	0.16	- 0.19	- 0.12	- 0.26
	P-value	0.464	0.552	0.691	0.547	0.549	0.672	0.415	0.304	0.246	0.425	0.264
Wind frequency	r_s	- 0.15	- 0.62	- 0.45	-0.01	0.03	- 0.10	0.01	- 0.23	- 0.39	- 0.30	- 0.41
	P-value	0.003	<0.001	<0.001	0.967	0.844	0.602	0.935	0.141	0.011	0.045	0.073
Wind run	r_s	- 0.09	- 0.23	- 0.17	0.09	- 0.05	- 0.08	- 0.13	- 0.06	- 0.31	- 0.28	- 0.26
	P-value	0.071	0.148	0.224	0.578	0.773	0.609	0.453	0.742	0.05	0.064	0.264

* Spearman correlation coefficients (r_s) were tested at a significance level of $P < 0.05$.

† Gene flow at 50 m was from the four ordinal directions (NE, NW, SE, SW) in 2015.

Table S4. AIC values and AIC differences (Δ) for the possible models to predict pollen-mediated gene flow (PMGF) under field conditions[*].

No.	Model[†]	*K*	*AIC*	Δ*AIC*	*LL*
1	GF~ Exp(1+dist) + Exp(1+dist*direc*yr)	35	8282.84	0	-4106.42
2	Exp(dist*direc*yr)+Exp(dist)	34	8329.424	46.58	-4130.71
3	Exp(dist*direc*yr)+Exp(dist*direc*yr)	33	8528.733	245.89	-4231.37
4	Exp(1+dist*direc*yr)	33	8700.491	417.65	-4317.25
5	Exp(dist)+Exp(dist+direc*yr)	18	9107.713	824.87	-4535.86
6	Exp(dist+direc*yr)+Exp(dist)	19	9404.469	1121.63	-4683.23
7	Exp(dist+direc*yr)+Exp(dist+direc*yr)	18	9465.676	1182.84	-4714.84
8	Exp(dist)+Exp(dist*wrun*yr)	9	10491.89	2209.05	-5236.95
9	Exp(dist*ws*yr)+Exp(dist+wrun)	10	10634.05	2351.21	-5307.03
10	Exp(dist)+Exp(dist*freq*yr)	9	10649.61	2366.77	-5315.81
11	Exp(dist)+Exp(dist*ws*yr)	9	10668.32	2385.48	-5325.16
12	Exp(1+dist)+Exp(dist*ws)	6	10702.53	2419.69	-5345.27
13	Exp(dist)+Exp(dist+wrun*yr)	6	10786.14	2503.3	-5387.07
14	Exp(dist)+Exp(dist*direc)	18	10886.93	2604.09	-5425.47
15	Exp(dist*direc)	17	10919.79	2636.95	-5442.9
16	Exp(dist+ws*yr)+ Exp(dist)	6	11032.06	2749.22	-5510.03
17	Exp(dist*freq*yr)	8	11041.99	2759.15	-5512.99
18	Exp(dist+direc)+Exp(dist+direc)	10	11115.02	2832.18	-5547.51
19	Exp(dist)+Exp(dist+ws*freq)	6	11202.11	2919.27	-5595.05
20	Exp(dist)+Exp(dist*wrun)	5	11202.82	2919.98	-5596.41
21	Exp(dist)+Exp(dist+freq*yr)	6	11248.12	2965.28	-5618.06
22	Exp(dist*wrun)+Exp(dist)	5	11264.35	2981.51	-5627.17
23	Exp(dist+direc)+Exp(dist)	10	11267.32	2984.48	-5623.66
24	Exp(dist)+Exp(dist*ws)	5	11303.57	3020.73	-5646.79
25	Exp(dist)+Exp(dist+direc)	10	11382.92	3100.08	-5681.46
26	Exp(dist*freq*yr)+Exp(dist*freq*yr)	8	11388.55	3105.71	-5686.28
27	Exp(dist*ws)+Exp(dist)	5	11388.61	3105.77	-5689.3
28	Exp(dist)+ Exp(dist+wrun)	4	11635.37	3352.53	-5813.68
29	Exp(dist+ws)+Exp(dist)	4	11689.66	3406.82	-5840.83
30	Exp(dist+freq*yr)+Exp(dist+freq*yr)	5	11743.33	3460.49	-5866.67
31	Exp(dist*ws*yr)+Exp(dist*ws*yr)	8	11819.4	3536.56	-5817.02
32	Exp(dist+freq*yr)	5	11952.81	3669.97	-5971.41
33	Exp(dist*wrun*yr)+Exp(dist*wrun*yr)	8	12096.03	3813.19	-6040.01
34	Exp(dist*freq)+ Exp(dist)	5	12145.59	3862.75	-6067.8

35	Exp(dist+ws)+ Exp(dist+ws)	4	12147.38	3864.54	-6069.69
36	Exp(dist+ws*yr)+Exp(dist+ws*yr)	5	12221.14	3938.3	-6105.57
37	Exp(dist+wrun*yr)+Exp(dist+wrun*yr)	5	12590.2	4307.36	-6290.1
38	Exp(dist*ws)+Exp(dist*ws)	4	12968.52	4685.68	-6480.26
39	Exp(dist*wrun)+ Exp(dist*wrun)	4	13164.19	4881.35	-6578.1
40	Exp(dist+wrun)+ Exp(dist+wrun)	3	13233.39	4950.55	-6613.7
41	Exp(dist*freq)+ Exp(dist*freq)	4	13290.39	5007.55	-6641.2
42	Exp(dist)+Exp(dist)	2	13345.23	5062.39	-6670.62
43	Exp(dist+freq)+Exp(dist+freq)	3	13345.8	5062.96	-6669.9

[*] *AIC* is the Akaike's Information Criterion calculated using $AIC = -2LL + 2K$; K is the number of parameters; LL is the maximized log likelihood.

[†] dist = distance from the pollen source; direc = directions of the pollen receptor blocks; GF = gene flow frequency; PMGF = pollen-mediated gene flow; ws = wind speed; freq = wind frequency; wrun = wind run (i.e., ws × freq); yr = year.
